# Awareness, attitudes, need and demand on replacement of missing teeth among a group of partially dentate patients attending a University Dental Hospital

**DOI:** 10.1186/s13104-017-2655-0

**Published:** 2017-07-27

**Authors:** Rasika Manori Jayasinghe, Janana Perera, Vajira Jayasinghe, Indika P. Thilakumara, Sumudu Rasnayaka, Muhammad Hanafi Muhammad Shiraz, Indra Ranabahu, Sanjeewa Kularatna

**Affiliations:** 1 Department of Prosthetic Dentistry, Faculty of Dental Sciences, University of Peradeniya, Peradeniya, Sri Lanka; 2 Australian Centre for Health Services Innovation, School of Public Health and Social Work, Queensland University of Technology, Brisbane City, Australia

**Keywords:** Awareness, Need, Demand, Prosthodontic options, Partially dentate

## Abstract

**Objective:**

Our objective was to assess awareness, attitudes, need and demand on replacement of missing teeth according to edentulous space, age, gender, ethnicity, educational level and socio-economical status of the patient.

**Results:**

76.2% of the study group was opined that the missing teeth should be replaced by prosthetic means. Majority were keen in getting them replaced mainly for the comfort in mastication. Although 77.9 and 32.9% were aware of the removable prostheses and implants respectively, only 25.2% knew about tooth supported bridges as an option of replacement of missing teeth. Participants’ awareness on tooth and implant supported prostheses is at a higher level. Participants’ opinion on need of regular dental visit was statistically significant when gender, ethnicity and education level were considered. The highest demand for replacement of missing teeth was observed in Kennedy class I and II situations in both upper and lower arches. Demand for fixed prostheses was significantly highest in Kennedy class II in upper and lower arches. In conclusion, although removable prosthodontic options are known to most of the patients, their awareness on tooth and implant supported prostheses is also at a higher level. The highest demand for replacement of missing teeth is by patients with Kennedy class I and II situations whereas Kennedy class II being the category with highest demand for fixed prostheses. We recommend that the location of missing teeth to be considered as a priority when educating patients on the most appropriate prosthetic treatment options. Dentists’ involvement in educating patients on prosthetic options needs to be improved.

## Introduction

Prosthodontic treatment depends on a variety of factors. The traditional approach resulted in a fairly uniform treatment option based on the fact that the missing teeth should always be replaced [1]. With the advancements and knowledge in dentistry and improvement in oral health with declining edentulousness in many countries, a higher number of people tend to keep more teeth until later in life [2]. However, of late, patient requirements such as esthetics and functional comfort are considered more important when attempting to replace missing teeth [3–5]. Although several prosthodontic options for the replacement of missing teeth are available, some researchers have highlighted that the acceptability of these options depend on the patient’s education, economy, cultural background as well as the age [6].

The methods discussed for evaluating the need for prosthetic management of partially dentate patients include the patients’ demand for treatment and the objective oral status of the patients [7]. Being the only institution in the country which is involved in both undergraduate and postgraduate dental education with an attached teaching hospital, the findings provide an accurate picture of the patients seeking treatment from a tertiary care dental hospital. The findings of this study will help to identify the level of awareness, attitudes and demand with regard to prosthodontic treatment options in partially dentate patients as the treatment options available in the government and private sector have also increased in the country. We also attempted to identify if there is a difference between the demand (patient’s perspective) and the need (the clinician’s perspective). Moreover, it was expected that this will also help prevent both under and overtreatment for a given patient.

## Main text

The main objective of the study was to assess the awareness, attitudes, need and demand for the replacement of missing teeth made by the participants. We also attempted to assess those four factors (awareness, attitudes, need and demand for replacement of missing teeth) specifically according to variables such as the location of the edentulous space, age, gender, ethnicity, educational level and the socio-economical status of the participant.

This cross-sectional study sample was selected from the screening clinic conducted every morning on weekdays at the Dental Hospital, Peradeniya during the years 2015 and 2016. An information sheet regarding this study was provided to all patients in their preferred language and informed written consent was obtained prior to the study.

Being a partially dentate patient and above 18 years of age were considered as the inclusion Criteria. Mentally retarded/disabled or physically handicapped patients and those from dental-related professions/occupations (dental surgeons, dental surgeons’ assistants, dental technical officers and dental students) and edentulous patients were excluded from the study sample.

Following the formula, N = z^2^p (1−p)/e^2^ and considering the fact that the population proportion for partially dentate patients (p) was not known, the safest choice was to use p as 0.5 (p = 0.5) for the calculation of the minimum sample size of 384 in this study.

All participants (425) were subjected to an interview and a pre tested and validated questionnaire in Sinhala and Tamil languages was administered. Demographic information was recorded by the subjects. The questionnaire (printed) consisted of open ended questions related to the knowledge, attitude, and awareness of the treatment options available for tooth replacement and the justification for selecting one option over another. All questionnaires were anonymous.

A clinical examination was carried out using a mouth mirror and a probe. Each patient was examined by two separate examiners who were specialists in prosthetic dentistry/senior house officers in the Department of Prosthetic Dentistry under the supervision of a specialist in prosthetic dentistry to avoid inter examiner variability. Every tenth patient was examined twice to minimize intra-examiner variability. The house officers were trained by specialists and specialists were also available in the clinic to address any problem regarding examination, diagnosis and available treatment options. The examination was used to evaluate the need for treatment and to select the suitable prosthetic treatment option for each patient following WHO basic methods and according to the Kennedy classification of each arch. The treatment options considered here were removable partial dentures, fixed tooth supported prostheses or implant supported removable/fixed prostheses.

Responses to all questions were collected and analyzed statistically using SPSS 17.0. The results were analyzed using logistic regression by considering age, gender, ethnicity, educational level and monthly income as independent variables. Ethical Clearance was sought from the Ethics review committee, Faculty of Dental Sciences, University of Peradeniya. (ERC/FDS/UOP/I/2015/18).

The mean age of the sample was 44.38 years and two-thirds of the respondents were females. Almost all participants (99.5%) were aware that they had missing tooth/teeth. Although their awareness on their partially dentate status correlated significantly to their level of education, it was non significant when gender was considered. (Table 1).

**Table 1:** Patients’ awareness of the partially dentate status according to the level of education

Characteristic	Number	Percentage	Number	Percentage	Significance
*Do you have any missing tooth/teeth?*	No	(n = 2)	Yes	(n = 423)	
Education level					
Primary education	1	50.0%	25	5.9%	
Up to grade 8	0	0.0%	38	9.0%	
Ordinary level (O/L)	0	0.0%	173	40.9%	p *=* *0.022*
Advanced level and above (A/L)	0	0.0%	147	34.8%	

When participants’ attitude towards the replacement of missing teeth was assessed, 76.2% were in favor of having missing teeth replaced (Table 2). However, the results were statistically non significant according to the variables we assessed. When questioned regarding the need of replacement, 101 out of 425 stated that there is no need of replacing lost teeth. Out of the remaining respondents who had a positive attitude towards the replacement of missing teeth (324), the majority claimed that the main aim of their replacement was the improvement of the masticatory ability. The highest percentage of the sample with a negative attitude towards tooth replacement was of the opinion that they did not need the replacement and the second highest reason was financial constraints (Table 3). Most of the participants (58.1%) felt that the replacement of both anterior and posterior teeth is equally valuable, whereas 26.6% thought that the replacement of anterior teeth is more important (Table 4). The results were statistically significant when monthly income was considered. (p = 0.013).

**Table 2:** Attitude towards the replacement of missing teeth in relation to age, gender, ethnicity, educational levels and the socio economic levels

*Do you think you need replacement when tooth/teeth are lost?*	No	(n = 101) (%)	Yes	(n = 324) (%)	p value	95% CI
Age category (years)						
16–20	3	3.0	11	3.4	p = 0.251	−0.001 to −0.005
21–40	44	43.6	111	34.4		
41–60	41	40.6	167	51.7		
61–80	13	12.9	34	10.5		
Gender						
Male	35	34.7	118	36.4	p = 0.946	−0.089 to −0.083
Female	66	65.3	206	63.6		
Ethnicity						0.049 to 0.102
Sinhala	93	92.1	291	89.8	p = 0.493	
Tamil	1	1.0	8	2.5		
Muslim	7	6.9	25	7.7		
Education level						
Primary education	10	9.9	16	4.9	p = 0.807	−0.041 to −0.053
Up to Grade 8	7	6.9	31	9.6		
O/L	41	40.6	132	40.7		
A/L	33	32.7	114	35.2		
Tertiary education	10	9.9	31	9.6		
Monthly income						
<10,000/= Rs	18	17.8	38	11.7	p = 0.101	−0.006 to −0.068
10,000/ = −20,000/= Rs	23	22.8	75	23.1		
20,000/= −30,000/= Rs	31	30.7	93	28.7		
30,000/= −40,000/= Rs	17	16.8	64	19.8		
>40,000/= Rs	12	11.9	54	16.7

**Table 3:** Participants reasons for not trying to replace missing teeth

*Reason*-*for no need of replacement when tooth/teeth is lost*	Number (total 101)
Financial	16
Did not feel it is needed	68
No time	3
Do not know about treatment options	7
Many reasons	7

24% of the participants were not keen in getting missing teeth replaced. The majority felt that they did not feel the need for the replacement and financial constraints also played a role in making the decision

**Table 4:** The comparative importance of the type of teeth to be replaced according to participants’ attitude

Independent variable	p value	95% CI
Age	0.695	−0.006 to 0.009
Gender	0.831	−0.224 to 0.180
Ethnicity	0.864	−0.193 to −0.162
Education level	0.485	−0.149 to 0.071
Monthly income	*0.013*	0.024 to 0.198

The majority of the patients were of the opinion that it is important to replace both anterior and posterior missing teeth

Results were statistically significant when monthly income was considered

When the participants were questioned regarding their plan towards the replacement of missing teeth, 68.7% displayed their preference to do so. Although 77.9% of the participants knew about removable prostheses for the replacement of missing teeth, only 25.2 and 32.9% knew about the tooth supported and implant supported prostheses respectively (Tables 5, 6, 7). The awareness on tooth supported fixed prostheses was statistically significant when age and educational level were considered.

**Table 5:** Participants’ awareness of different types of prostheses used to replace missing teeth

Awareness on type of prostheses	Number	Percentage of population
Removable prostheses	331	77.9
Tooth supported bridges	107	25.2
Implant supported prostheses	140	32.9

About 78% of the participants were aware of removable prostheses as a mode of replacement of missing teeth. However, awareness of tooth supported fixed prostheses was less than implant-supported prostheses

**Table 6:** Participants’ awareness on tooth supported fixed prostheses according to different variables

Independent variable	p value	95% CI
Age	*0.030*	−0.000 to 0.007
Gender	0.874	−0.093 to 0.079
Ethnicity	0.635	−0.057 to 0.094
Education level	*0.024*	0.007 to 0.101
Monthly income	0.056	0.000 to 0.073

Participants’ awareness on tooth supported bridges was statistically significant when age and educational level were considered

**Table 7:** Participants’ demand when they were educated on different types of prostheses available

Demand for different prostheses	Number	Percentage of participants
No prosthesis	44	10.4
Removable prosthesis	117	27.5
Tooth supported fixed prosthesis	72	16.9
Implant supported fixed prosthesis	135	31.8
Tooth/implant supported fixed prosthesis	57	13.4

Once the knowledge on different prosthodontic options was provided, more patients requested tooth and implant-supported prostheses than removable ones. Surprisingly, 10% did not demand any type of replacement

The participants’ awareness of regular dental visits was also assessed according to variables such as gender, ethnicity and educational level and the results revealed that it is statistically significant when gender, ethnicity and the educational levels were considered. (Table 8).

**Table 8:** Participants’ awareness of the need for regular dental visits

Do you think that a regular dental visit is needed for everybody?	No	(n = 37) (%)	Yes	(n = 386)	p value	95% CI
Age category (years)						
16–20	2	5.4	12	3.1		
21–40	11	29.7	144	37.4		
41–60	16	43.2	191	49.6	p = 0.473	−0.003 to 0.001
61–80	8	21.6	38	9.9		
Gender						
Male	21	56.8	131	33.9	p *=* *0.009*	0.018 to 0.131
Female	16	43.2	255	66.1		
Ethnicity						
Sinhala	37	100.0	346	89.6		
Tamil	0	0.0	9	2.3		
Muslim	0	0.0	31	8.0	p = *0.033*	0.004 to 0.104
Education level						
Primary education	3	8.1	23	6.0		
Up to grade 8	9	24.3	29	7.5		
O/L	16	43.2	156	40.4	p = *0.025*	0.004 to 0.066
A/L	7	18.9	139	36.0		
Tertiary education	2	5.4	39	10.1		
Monthly income						
<10,000/= Rs	8	21.6	48	12.4		
10,000/= −20,000/= Rs	7	18.9	90	23.3		
20,000/= −30,000/= Rs	13	35.1	111	28.8	p = 0.702	−0.020 to 0.029
30,000/= −40,000/= Rs	4	10.8	77	19.9		
>40,000/= Rs						

Females were more aware of the need for regular dental visits by all individuals and the results were statistically significant

Participants of Sinhala nationality and with higher levels of education were more aware of the fact and the results were statistically significant

When the demand for the replacement of missing teeth was analyzed according to the position of the edentulous region in upper arch and lower arch, the results revealed that participants with Kennedy class I and II have the highest demand and this demand level was statistically significant. (Tables 9, 10) The demand for fixed prostheses was highest in Kennedy class II in upper and lower arches and the results were statistically significant. All Kennedy class IV patients were interested in fixed prostheses as the method of replacement.

**Table 9:** The demand for the replacement of missing teeth according to the position of saddle in upper arch

Upper arch Kennedy	None	RPD (removable partial dentures)	TS (tooth supported) bridges	Implants	TS bridges and implants	Percentage requested fixed prosthesis	Percentage requested replacement
CI (Class I)	0	22	3	6	4	37	100
CII (Class II)	6	16	14	14	8	62	98
CIII (Class III)	32	61	37	2	9	34	77
CIV (Class IV)	0	2	1	1	0	50	100

Individuals of Maxillary Kennedy class I, II and IV were more interested in getting missing teeth replaced than class III individuals. A higher number of class II and IV individuals were keener on fixed options for replacing missing teeth than the other two classes

**Table 10:** The demand for the replacement of missing teeth according to the position of saddle in lower arch

Lower arch Kennedy	None	RPD	TS bridges	Implants	TS bridges and implants	Percentage requested fixed prosthesis	Percentage requested replacement
CI	6	36	8	12	8	40	91
CII	7	28	13	28	12	66	92
CIII	27	45	40	6	5	23	71
CIV	0	0	1	1	0	100	100

Individuals of Mandibular Kennedy class I, II and IV were more favorable towards the replacement of missing teeth than class III individuals. All class IV participants were interested in fixed prostheses as the option of replacement

## Limitations

The patients’ attitudes and demand towards the replacement of missing teeth might be different from the clinicians’ assessment. Although prosthodontists consider factors such as the preservation of natural teeth and the maintenance of periodontal health as priority, patients tend to prioritize comfort in mastication and improvement of esthetics. Therefore, it is vital to investigate patients’ awareness, need and demand on prosthodontic treatment options. To the best of our knowledge, ours could be the first Sri Lankan study which assessed those variables on prosthodontic options.

Our finding that 99.5% of the respondents in the sample were aware of their partially dentate status, corresponded with other reports which have shown a high level of awareness (95.93%). However, some studies have found a slightly significant influence of gender, which was not revealed in our study [8]. The majority of our patients were of the opinion that it is more important to replace anterior teeth and this finding is consistent with the previous studies [7].

When the participants’ attitude towards the replacement of missing teeth was assessed, 76.2% of the sample showed positive attitudes, and this value is almost double the value reported by Reddy et al. [8] in Saudi Arabia in 2016. However, both studies failed to identify any statistically significant difference when attitude was assessed against the gender and the educational level of the participants. Most of the participants with negative attitudes had responded that they didn’t feel that it is required to get the missing teeth replaced and the second most frequent reason given for the negative attitude was financial constraints. However, an Indian study identified financial factors as the main barrier in the decision for the replacement of missing teeth [9].

Out of 425 participants, 32% was aware of implants as a mode of replacement. This finding corresponds to reports by previous authors [10] and the percentage we reported is higher compared to multiple studies in the Asian population [8, 11]. This disparity could be attributed to the higher standards of health and education in the country compared to the neighbouring countries in the region.

Moreover, we observed a very high participants’ preference towards fixed prostheses supported by implants or teeth (62%).The report of the study by Al-Quran et al. in [11] also confirms our results as only 34% preferred removable prostheses as an option. However, a similar study in Saudi Arabia highlighted that around 50% of the sample were keen on removable partial dentures while 25% preferred tooth supported fixed prostheses, and surprisingly, none opted for implants [8]. The difference in our outcome could be explained with the higher literacy level in the country as our results were positively associated with participants’ educational level and due to the use of newer mass media by the general public.

Our study claims that patients’ demand for fixed prostheses is at a higher level when patients were educated on the issue and that dentists have a duty to spend time on educating patients regarding available prosthetic options. This claim is supported by the fact that most of the patients (about 34%) highlighted that their source of information on prosthodontic options was friends or relatives. However, a study by Mukatash et al. [7] carried out in Jordan found that the major source of information for patients is dentists. Both studies reveal that mass media does not play a significant role in the education on prosthodontic aspects of dental health.

The statistically significant highest demand for fixed prostheses in Kennedy class II in upper and lower arch could be due to difficulty in mastication (which participants identified as the most important reason for replacing missing teeth) and discomfort with free end saddle removable prostheses. The fact that Kennedy class I and II have the highest overall demand for prosthetic replacement of missing teeth confirms that patients’ concern for the improvement of mastication plays an important role in this high demand. The assessment of demand for different types of prostheses according to the location of the edentulous space is important in order to identify the most appropriate type of prosthesis for the patients attending dental clinics.

We consider the fact that more than 90% of the participants were aware that regular dental visits are required in order to maintain optimum oral and dental health of an individual to be a positive factor.

In conclusion, the majority of the patients were keen on getting missing teeth replaced mainly for comfortable mastication. While the removable prosthodontic options are known to most of the patients, their awareness on tooth and implant supported prostheses is also at a higher level. The highest demand for the replacement of missing teeth was made by patients with Kennedy class I and II situations while Kennedy class II was the category with the highest demand for fixed prostheses.

We recommend that the location of missing teeth be considered as a priority when educating patients on the most appropriate prosthetic treatment options. Dentists’ involvement in educating patients on prosthetic options needs to be improved.

## Abbreviations

O/L: ordinary level
A/L: advanced level
RPD: removable partial dentures
TS: tooth supported
CI: class I
CII: class II
CIII: class III
CIV: class IV
